# Diseases of the Digestive Organs in Infancy and Childhood

**Published:** 1886-07

**Authors:** 


**Diseases of the Digestive Organs in Infancy and Childhood,** with chapters on the investigation of diseases, and on the general management of children. By LOUIS STARR, M. D., Clinical Professor of Diseases of Children, in the Hospital of the University of Pennsylvania; with illustrations. Philadelphia: P. Blakiston, Son & Co. 1886.

This book ought to have a large sale, for it admirably treats of a class of diseases constituting a large proportion of the ailments of childhood, and to which the standard text-books devote but meagre space. The author wisely recognizes that the administration of drugs is less important than attention to the general regimen, and he gives prominence to dietetics, artificial digestion, and the regulation of clothing — vital points upon which the young practitioner is too often illy instructed. The chapter on the investigation of diseases is a good one, and though not necessarily connected with the subject of the book, will increase its value to many practitioners.

